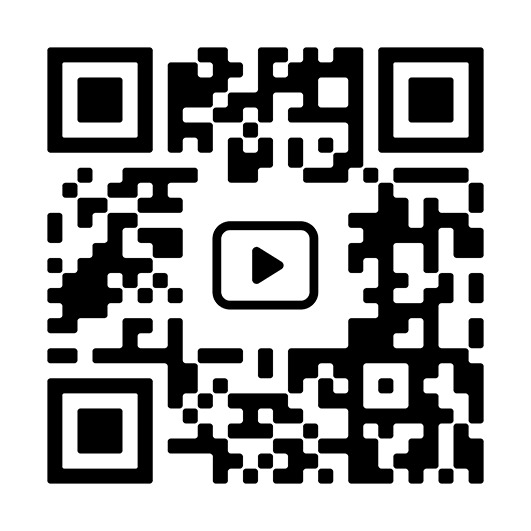# Surgical steps of total laparoscopic hysterectomy: Part 1: Benign disease by the European Society for Gynaecological Endoscopy (ESGE)^1^

**Published:** 2019-06

**Authors:** Working group of ESGE, G Pados, S Becker, R Rovira Negre, B Rabischong, H Ferreira, C Rossitto

**Affiliations:** ESGE Central Office Diestsevest 43/0001 3000 Leuven, Belgium.

**Keywords:** Laparoscopic hysterectomy, benign disease, operative outcome, surgical steps, minimally invasive surgery

## Abstract

**Background:**

Laparoscopic hysterectomy, which is associated with well-documented benefits compared to an abdominal approach, should be the surgical treatment choice for certain gynaecological conditions. The aim of this document is to provide recommendations on the technical aspects of this procedure.

**Materials and methods:**

The European Society for Gynaecological Endoscopy (ESGE) has established a working group with the goal of standardizing the surgical steps of total laparoscopic hysterectomy for benign disease, which is one of the most commonly performed major surgical procedures in Gynaecology.

**Results:**

A properly equipped surgical theatre and the correct positioning of both the laparoscopic tower and the surgeons are of utmost importance and insure that the surgical procedure can be performed uneventfully.The incorporation of standardized surgical steps in routine practice should help to ensure an optimal surgical outcome based on the available scientific evidence as reviewed by expert surgeons.

**Conclusions:**

The description of the surgical steps of total laparoscopic hysterectomy and its adoption by minimally invasive gynaecologists will make this approach safer.

## Introduction

Hysterectomy is the most frequently performed major surgical procedure in gynaecology (Aaarts et al., 2015) and can be performed for benign and malignant indications. Approximately 90% of hysterectomies are performed for benign conditions (Flory et al., 2005). The indications are mainly leiomyomas leading to abnormal uterine bleeding (40%), endometriosis/adenomyosis leading to dysmenorrhea, dyspareunia, abnormal uterine bleeding (18%) and pelvic prolapse (14%; ACOG, 2017).

The above-mentioned benign diseases can be approached by conservative treatment, medical or surgical operation or by definitive surgery, which is hysterectomy. Since this is the only approach that can potentially provide permanent relief of symptoms, a remarkable proportion of women will definitely decide to have their uterus removed. The selection of the route for hysterectomy for benign disease can be influenced by the size of the uterus, the concomitant pathology, the need for concurrent procedures, surgeon skills and training, technological environment and support of the operating room, scheduled or emergent nature of the particular case and finally the patient’s preference. The approaches to hysterectomy can be broadly classified into four distinct categories: abdominal hysterectomy (AH), vaginal hysterectomy (VH), laparoscopic hysterectomy (LH) and robotic-assisted hysterectomy (RH).

First performed in January 1988 by Harry Reich et al, LH stimulated a widespread interest in the laparoscopic approach to hysterectomy (Reich et al., 1989). Soon after, this technique was claimed by enthusiastic pioneers and began to be scientificallyreviewed. Evidence-based studies have shown that LH is a better alternative to AH in terms of lower postoperative morbidity, early return to normal activities, better cosmetic results and lower costs and improved quality of health care (Aaarts et al., 2015). At the same time, the laparoscopic approach introduces new categories of complications, in addition to the possible known ones observed in abdominal and vaginal hysterectomy.

Therefore, the aim of this document is to offer a better insight and a detailed description of the surgical steps of laparoscopic hysterectomy, in order to better govern this innovative approach for the benefit of patients and provide recommendations covering the technical aspects of this approach.

## Materials and methods

### 

The composition of the working group was done after the initial submission of the initiative by the coordinator (GP) to the ESGE Advisory Board meeting on October 1, 2016 and included invited “minimally invasive surgery” experts. The project was then approved by the Executive Board of ESGE and shortly thereafter a live meeting was organized among the members of the working group. Then, a draft was distributed in two rounds to seek the views of the members of the working group, and the final document was prepared on the basis of the comments. The final draft was redistributed for final comments and approval by all ESGE members and was exposed for an open review on the Society’s website. The final document, after taking into account all comments, was reviewed and approved by the ESGE Executive Board.

### I. Operation theatre set-up and operative access

#### 

A properly equipped operation theatre and the correct positioning of the laparoscopic tower and surgeons are key factors to ensure that the surgical procedure can be performed uneventfully (Knight, 2018; Reich, 2007; Velemir, 2009).

#### 1. Patient, material and surgical team organisation

##### 1.1. Patient positioning:

- The patient must be placed in lithotomy with the legs spread apart in little ventral flexion. This position allows lateral movement of the uterine manipulator.
- The buttocks should be placed slightly above the edge of the operating table and this position facilitates uterine manipulation.
- The arms must be placed along the patient’s body to avoid a brachial plexus injury.
- The use of shoulder braces may be considered, especially in cases where a pronounced Trendelenburg position is needed, although it has been associated with brachial plexus injuries. Alternatively, a specific foam or even a mattress that is taped to the operating table can be used.
- The patient must not slide cephalad during Trendelenburg position.

##### 1.2. Material and surgical team organisation:

- The laparoscopic tower is usually placed under the patient’s right feet or in the middle of the legs, which allows the surgeon and the first assistant a clear vision. In cases of multiple monitors, they must be placed in front of each member of the surgical team, allowing a clear vision and an ergonomic neck position. Whenever possible, the assistant holding the uterine manipulator should also have visual access to the surgery.
- The material (cables, irrigation), must be placed in an organized state to avoid cables mixing during the surgery.
- When the surgeon is right-handed, he or she will normally work from the left side of the patient. Then, the first assistant will be placed on the right side and the second assistant will be placed between the legs. Some right-handed surgeons prefer to be to the patient’s right to allow ease of suturing cross table with the right hand.

#### 2. Abdominal access: Pneumoperitoneum creation

##### 2.1. Before pneumoperitoneum creation:

- The table must be placed in neutral position (in case of a pronounced Trendelenburg position before pneumoperitoneum creation, the risk of vascular injury increases).
- The procedure is performed under general endotracheal anaesthesia, while the nasogastric tube, whenever it is used, must be checked to be in place.
- The pneumoperitoneum circuit must be checked.
- Some surgeons prefer to gain access through an abdominal wall that is exactly parallel to the floor. To achieve this “even plane”, the patient will have to be placed at fifteen degrees Trendelenburg. This should to be taken into account when inserting the Veress needle.

##### 2.2. Achieving intraperitoneal access using a recognized method:

- In the case of Veress needle access, its orientation will depend on the patient’s degree of obesity. The more obese a patient is, the more perpendicular the access should be.
- Before Palmer point access, the surgeon must ensure that the nasogastric tube is working and that the stomach is perfectly decompressed. Splenomegaly must be ruled out.
- Alternative Access-Techniques include open laparoscopic access, use of optical trocars or direct trocar access.
- In the case of the Veress needle, the CO2 gas should be turned on and the monitor should be looked at to check for two readings, pressure and flow.
- If it is in the correct position, the intra-abdominal pressure should be <15 mm and it should fall soon after, with muscle relaxation at <10 mm.
- When the pressure is kept high (18 – 20 mm), the needle may be in the omentum, which can be dislodged by slightly withdrawing and gently shaking the needle tip.
- The correct position should be confirmed by observing that the flow should be approximately 1 - 1.5 l/min, depending on the diameter of the Veress needle.
- A persistent high pressure/low flow situation usually means that the tip is located on the abdominal wall, which indicates reinsertion.

#### 3. Inspection of the abdominal cavity

##### 3.1. Performing a diagnostic laparoscopy:

- Once the pneumoperitoneum is created (12-15 mmHg), entrance placement must be checked, looking for possible intestinal, vascular injuries or pre-pneumoperitoneum.
- All four quadrants of the abdominal cavity must be examined, including the liver and diaphragm.

##### 3.2. Optimal exposure of the pelvis:

- The Trendelenburg position is requested until the small bowel can be completely moved out of the pelvis.
- The optimal Trendelenburg position will allow the visualization of the promontory and the right ureter crossing the external iliac artery.
- The table must be placed as low as possible, allowing an ergonomic position. In case this is not achieved, the platforms should be used by the surgical team.

#### 4. Trocar insertion

##### 4.1. Checking the anatomy before trocar insertion:

- Reference points should be defined: anterior superior Iliac spine – navel – midline.
- Obesity status and “Danger” level at entry must be accessed.
- Scar selection entry technique (Direct – Veress – Open – Optical Trocar – Palmer technique) should be examined.
- It is advisable to check the size of the structures to be removed before trocar insertion.
- Trocar placement must be done taking into account the size and the situation of the structure that is going to be removed.

##### 4.2. Epigastric vessels situation and ergonomic trocar insertion:

- The location of the epigastric vessels and the umbilical artery must be checked before trocar insertion in order to avoid injury. The lateral trocars should be inserted laterally into the inferior epigastric vessels, which can be visualized in most cases through the peritoneum. If this is not the case, the rule is to insert the trocars lateral to the rectus muscle. Trans-illumination does not delineate the epigastric arteries well.
- Adhesiolysis occurs before trocar insertion to ensure its optimal placement.
- An adequate number of working trocar insertions is usually three. Make sure the assistant uses both hands.
- The optimal trocar orientation is 90° from the abdominal plane. Trocar placement must allow free movements of the instrument.
- Trocar placement in ergonomic situation will allow an optimal triangulation.
- General rule: High and Lateral

##### 4.3. Checking trocar insertion injuries:

- Possible trocar insertion injuries (abdominal, bowel, vascular, etc) should be checked, even if it requires mobilizing the omentum and the bowel.

#### 5. Inspection of the pelvis

##### 5.1. Pelvis exposure:

- Pelvis exposure, mobilisation of the small bowel, sigmoid and performing adhesiolysis, if necessary, are carried out.

##### 5.2. Uterine and adnexa inspection:

- The different sides of the uterine anatomy and the adnexa anatomy should be inspected.

##### 5.3. Uterine manipulator insertion: (2nd assistant)

- There should be an adequate uterine manipulator insertion without lesions to the surrounding structures.
- Comment: Some surgeons place trocars routinely before a laparoscopic access is obtained. Accessory trocar placement must only be done under visualization.

##### 5.4. Checking for adequate uterine manipulator insertion:

- The uterine manipulator must be placed deep enough in the uterine cavity to allow maximum uterus mobility.

##### 5.5. Checking the anatomy around the internal genitalia:

- The pouch of Douglas and ovarian fossa should be checked.

##### 5.6. Checking ureters:

- The path of both ureters in the pelvis should be checked. At this point, it is advisable to divide the congenital adhesions between the sigmoid colon and the lateral pelvic wall, to facilitate the exposure of both the left ureter and the ipsilateral infundibulopelvic ligament.

### II. Surgical steps

#### 

**Figure 1:**
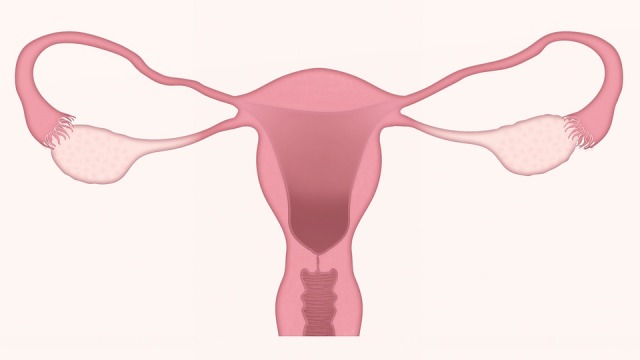
Schematic total laparoscopic hysterectomy.

Different surgeons will take different approaches to hysterectomy. The same surgeon can use different approaches for different clinical situations. The most important variants are discussed (Kondo, 2011; Einarsson et al., 2009; Thoma et al., 2007; Sandberg et al., 2017) (Table 1 and Figure 1).

**Table I:** — Surgical steps of Total laparoscopic hysterectomy.

1. Division of the round ligaments
2. Treatment of the adnexa
3. Dissection of the vesico-uterine space
4. Opening posterior peritoneum
5. Uterine vessels division
6. Colpotomy
7. Uterus retrieval
8. Vaginal closure
9. Hemostasis and inspection
10. Trocar removal and skin suturing

#### 1. Start of surgery

Uterine manipulator:

- The uterine manipulator is pushed cranially, lateral to the opposite side of the dissection in an intermediate position (no ante- or retroversion) (2nd assistant).
- The first assistant catches the round ligament and pulls towards his/her side (1st assistant).

Hysterectomy basically requires the coagulation of four vessels (two uterine arteries, two utero-ovarian vessels). Surgery begins in the cornual region. Here, three anatomical structures are inserted into the uterus and connected to other different structures:

- Round ligament (Lig. Rotundum)
- Fallopian tube
- Ovarian ligament (Lig. Ovarium Proprium)

All these structures need to be coagulated and cut. In other words, anatomy will vary considerably from one case to another, as will surgical approaches. Furthermore, different approaches are described as follows:

Option 1.1: Division of the round ligaments (left and right)

1.1.Coagulation and dissection of the round ligament:

- It is recommended that this action be performed at the level of the avascular triangle of the broad ligament, limited laterally by the iliac vessels and medially by the adnexa vessels.
- It is important to perform it sufficiently away from the uterus, at the level of the umbilical artery (Tips & Tricks).

Reason: Retrograde bleeding from the uterine vessels that retract into the uterus should be avoided.

Option 1.2: Start of surgery at the ovarian ligament

Coagulation and dissection of the ovarian ligament was followed by coagulation and dissection of the round ligament (1 cm away from the uterine insertion), which was followed by the coagulation of the fallopian tube insertion into the uterus, and followed by direct coagulation of the utero-ovarian anastomosis.

1.3. In the case of leaving the ovaries in situ, prophylactic resection of the tubes was considered.Both options will lead to a division of both folds of the broad ligament – Option 1 closer to the pelvic sidewall (preferred option if a complete dissection of the pelvic sidewall is planned) – Option 2 closer to the uterus (preferred option if the pelvic sidewall will not be entered).

Division of both folds of the broad ligament:

- This action will allow CO2 entrance into the retroperitoneal space and help to separate the anterior and posterior fold of the broad ligament (Tips & Tricks).

#### 2. Treatment of the adnexa: Coagulation and section of IP ligaments or utero-ovarian ligaments (left and right)

##### 2.1. Uterine manipulator:

- The uterine manipulator is pushed cranially, lateral to the opposite side of the dissection in an intermediate position (no ante- or retroversion) (2nd assistant).

##### 2.2. IP ligament or utero-ovarian ligament exposition:

- It should be performed without damaging the surrounding tissues.
- There should be a high risk of bleeding from venous IP-vessels.

##### If fenestration is performed:

##### 2.3. Exposure of the grey area of the posterior leaf of the broad ligament.

##### 2.4. Opening a wide peritoneal window in the broad ligament.

##### 2.5. Checking if the ureter is far from the dissection area. A good way of identifying the ureter is to look for it at the point where the iliac arteries bifurcate, since the ureter crosses the lateral to the medial.

##### If fenestration is not performed:

##### 2.6. Checking the ureter by trans-peritoneal visualization.

##### 2.7. Coagulation of IP ligament or utero-ovarian ligament:

- It must be performed with an adequate energy source or suture.
- Insufficient coagulation of infundibulopelvic ligament (i.e. of ovarian vessels) should be avoided at all cost: the bleeding vessels will retract into the retroperitoneum, requiring a major retroperitoneal dissection for secondary haemostasis – at very high risk for ureteric lesion.

##### 2.8. Section of the IP ligament:

- The adnexa should be lifted without undue tension by the assistant and the coagulation of the IP ligament should be performed immediately below the ovarian contour, to avoid the thermal spread to the ureter
- Consider it at an angle of 90° without bleeding.

#### 3. Dissection of the vesico-uterine space

##### 3.1. Uterine manipulator:

- The uterine manipulator is pushed cranially, and slightly lateral to the opposite side where the dissection begins (2nd assistant).

##### 3.2. Opening of the anterior fold of the broad ligament:

- The anterior fold of the broad ligament is opened with dissection and coagulation towards medial vesical line.
- The vesico-uterine junction is identified as a white line firmly attached to the uterus, around 2-3 cm between the bladder and the uterus. The initial incision must be performed caudal to the white line.
- The dissection starts from the left side. It is very important that the first assistant pulls the round ligament of the uterus cranially and slightly to his/her side (1st assistant).

##### 3.3. Uterine manipulator:

- The uterine manipulator is pushed cranially, in a central position and slightly in retroverted position (2nd assistant).

##### 3.4. Section of the peritoneum down to the uterine segment:

- This section must be performed towards the uterine segment.
- It is recommended to open only the pre-vesical peritoneum. This helps to avoid vesical damage (Tips & Trick)s.

##### 3.5. Visualization and extraction of the bladder:

- The surgeon shows the first assistant where the bladder is by pushing it and the first assistant must grasp the bladder at the midline, applying an anterior-superior traction.

##### 3.6. Opening of the vesico-uterine space:

- In anatomically easy cases, this can be performed in the midline until the cervical margin is exposed.
- In anatomically difficult cases (after previous Caesarean sections), a lateral approach is usually recommended.
- The surgeon identifies the vesico-vaginal space and dissects this space in caudal direction with dissection and superficial coagulation, without damaging the bladder. Also, since fat belongs to the bladder, it is recommended to stay above the fat in order to avoid bladder injuries.
- The superficial layer of the vesico-uterine ligament is shown due to the traction of the bladder towards the anterior abdominal walls. Bladder pillars must be coagulated and cut on both sides.
- Some uterine manipulators have a colpotomy cup fitted and the cup outline is visible throughout the surgical procedure (e.g., Hohl manipulator). The cup outline helps identify the vaginal fornix and limits the amount of dissection of the bladder. This means that if such a manipulator is used, the bladder pillars does not need to be divided into normal anatomy.

#### 4. Opening of the posterior peritoneum (left and right)

##### 4.1. Uterine manipulator:

- The uterine manipulator is pushed anteriorly and cranially, and slightly lateral to the opposite side where the dissection begins (2nd assistant).

##### 4.2. Dissection of the posterior leaf of the broad ligament:

- Dissection, coagulation and section of the posterior peritoneum are performed towards the utero-sacral ligaments.
- Coagulation and section of the utero-sacral ligaments are not always necessary, but are recommended in larger uterus, because they help in uterine mobilization (Tips & Tricks).
- When using a uterine manipulator with a cap, colpotomy is usually possible cranial to the uterosacral ligaments. With this technique, they remain intact and do not need to be dissected at all, possibly preserving their (unclear) function with regard to the remaining vaginal vault.
- In cases with diseased peritoneum of the ovarian fossa (i.e., endometriosis – adhesions), the ureter should be recognized and dissected-free, possibly reaching the level of the ureteric channel (Tips and Tricks)

#### 5. Uterine vessels division

##### 5.1. Uterine manipulator:

- The uterine manipulator is pushed cranially, lateral to the opposite side of the dissection in an intermediate position (no ante- or retroversion). It is important to allow sufficient vision from the anterior, posterior and uterine side (2nd assistant).
- The first assistant catches the round ligament and pulls cranially and towards his/her side (1st assistant).

##### 5.2. Optimizing the exposure of the uterine vessels.

##### 5.3. Skeletonization of the uterine vessels:

- It is performed at the ascending portion of the uterine artery.
- The posterior peritoneum must be dissected, coagulated and sectioned towards the utero-sacral ligaments.

##### 5.4. Identifying the ureter before coagulation and the section of the uterine vessels:

- It is performed especially if the anatomy is distorted.

##### 5.5. Coagulation of the uterine vessels:

- It is done using the available energy source (the application of energy should be as short as possible) or by suturing.

##### 5.6. Transection of the uterine vessels:

- It should be performed in the ascendent portion with the help of the uterine manipulator (higher than the level of the colpotomizer).

##### 5.7. The cervical attachments of the paracervix should be divided.

During the intrafascial technique, a transverse incision is made through the fascia slightly below the level of the internal orifice of the uterus (or internal os) and the fascia is freely dissected with a probe.

#### 6. Colpotomy:

##### 6.1. Uterine manipulator:

- The uterine manipulator is pushed cranially. The vaginal occlusion system of the uterine manipulator must be in place (2nd assistant).

##### 6.2. Cervico-vaginal identification

##### 6.3. Dissection of the vaginal ring:

- Non-interposed elements around the vaginal fornices should be checked and a complete dissection if necessary.

##### 6.4. Colpotomy:

- An appropriate energy source was used for circumferential colpotomy. The colpotomizer must be placed in the same direction as the incision that helps the opening of the vagina by the surgeon (2nd assistant).

##### 6.5. Technical advice:

Consider starting colpotomy posteriorly (6 o’clock position), going left and right from 6 to 12 o’clock (towards “anterior”) and finishing at the 12 o’clock position, while avoiding excessive coagulation in the vaginal vault to ensure a good healing.

#### 7. Uterus retrieval:

##### 7.1. Specimens are retrieved vaginally, when possible or morcellation with or without containment techniques as applicable.

##### 7.2. The vagina occlusion was performed to restore pneumoperitoneum.

#### 8. Vaginal closure:

##### 8.1. The vaginal vault is sutured with interrupted or continuous sutures, laparoscopically or vaginally.

There is now a high-quality evidence from a randomized trial that closing the vaginal vault vaginally increases vault dehiscence and wound complications (Uccella et al., 2018).

##### 8.2. Vaginal suturing includes sufficient width of vaginal mucosa and fascia.

##### 8.3. The suture includes both utero-sacral ligaments for pelvic support.

NOTE: It is not evidence-based, but is supported by a panel group of experts on the H-OSATS scale: Utero-sacral ligament inclusion potentially increases the risk of ureteric injury (kinking).

#### 9. Haemostasis and inspection:

##### 9.1. The pelvis should be irrigated and aspirated (intra-abdominal pressure reduced to e.g. 9 mmHg)

##### 9.2. Vascular pedicles, vaginal vault, ureters and bladder should be checked.

##### 9.3. The surrounding structures should be checked for no damages.

##### 9.4. Cystoscopy is performed with Indigocarmine if a ureteral lesion is suspected.

NOTE: Indigocarmine is no longer available in many countries.

Methylene blue was strongly disliked by Anaesthesiologists and was not as effective.

If the ureteric injury is a concern, we recommend dissecting out the ureter if the gynaecologist has this competence. Alternatives include performing a retrograde ureterogram with the assistance of a urologist or even injecting methylene blue into the bladder diluted in saline (180-220 ml).

##### 9.5. Performing cystoscopy if vesical lesion is suspected.

#### 10. Trocar removal and skin suturing:

##### 10.1. Trocar removal under direct vision and examining haemostasis.

##### 10.2. Totally evacuating pneumoperitoneum.

##### 10.3. Suture fascia of trocars ≥ 10 mm.

Considering a local subcutaneous anaesthesia for the reduction of postoperative pain.

##### 10.4. Closing skin incisions.

## Conclusions

The available evidence indicates that laparoscopic hysterectomy should be considered the preferable alternative to abdominal hysterectomy in those patients in whom the vaginal approach is not indicated. Laparoscopic surgery, which is associated with proven benefits, has been adopted by gynaecologic surgical specialists as an effective surgical approach for the removal of the uterus. The standardisation of the surgical technique, the clinical practice guidelines and the detailed description of the surgical steps of LH are intended to make this approach safe and to ensure a favourable outcome for all patients.

## Video scan (read QR)

Supplementary video 1: Division of the round ligaments (Title).

1.1. Coagulation and dissection of the round ligament (subtitle) V1.1

1.2. Coagulation and section of the round ligament (using a sealing device) (subtitle) V1.2

Supplementary video 2: Treatment of the adnexa.

2.1. Coagulation and section of the infundibulo-pelvic ligament/suspensory ovarian ligament (subtitle) V2.1

2.2. Opportunistic salpingectomy (subtitle) V2.2

2.3. Coagulation and section of the utero-ovarian ligament (subtitle) V2.3

Supplementary video 3: Dissection of the vesico-uterine space.

3.1. Opening the vesico-uterine space (after C-section) pulling the bladder (subtitle) V3.1

3.2. Opening the vesico-uterine space with large dissection exposing vaginal tissue (subtitle) V3.2

3.3. Opening the vesico-uterine space using sealing instrument (subtitle) V3.3

Supplementary video 4: Opening posterior peritoneum.

4.1. Dissecting the posterior leaflet of broad ligament exposing the uterine vascular pedicle (subtitle) V4.1

4.2. Opening posterior peritoneum developing uterine mobility and better exposure of the uterine vessels (subtitle) V 4.2

Supplementary video 5: Uterine vessels division.

5.1. Isolating the uterine vessels (artery and vein) applying bipolar energy causing desiccation and then cutting the vessels (subtitle) V 5.1

5.2. Uterine vessels coagulation and section using a vessel sealing device (subtitle) V 5.2

Supplementary video 6: Colpotomy.

6.1. Opening the vaginal cuff using monopolar hook and the uterine manipulator (subtitle) V 6.1

6.2. Opening the vaginal cuff using monopolar hook (subtitle) V 6.2

Supplementary video 7: Uterus retrieval.

Uterus extraction using the uterine manipulator and careful mobilization by the 2nd assistant (subtitle) V 7

Supplementary video 8: Vaginal closure.

8.1. Vagina closed by intra-corporeal suturing (subtitle) (V8.1)

8.2. Vaginal closed by extra-corporeal suturing (subtitle) (V8.2)

Supplementary video 9: Hemostasis and inspection.

9.1. Careful inspection of hemostasis irrigating to exclude any possible bleeding (subtitle) (V9.1)

9.2. Meticulous hemostasis and lavage (V9.2)

**Figure:**